# The solute carrier transporters (SLCs) family in nutrient metabolism and ferroptosis

**DOI:** 10.1186/s40364-024-00645-2

**Published:** 2024-09-02

**Authors:** Li-Li Sun, Hai-Yan He, Wei Li, Wei-Lin Jin, Yi-Ju Wei

**Affiliations:** 1 School of Life Science, Shandong First Medical University & Shandong Academy of Medical Sciences, Jinan, Shandong 250117 China; 2 Medical Science and Technology Innovation Center, Shandong First Medical University & Shandong Academy of Medical Sciences, Jinan, Shandong 250117 China; 3 Department of Pharmacy, Sir Run Run Shaw Hospital, School of Medicine, Zhejiang University, Hangzhou, Zhejiang P. R. China; 4 Division of Hematology and Oncology, Department of Pediatrics, Penn State Cancer Institute, Penn State College of Medicine, Hershey, PA 17033 USA; 5 Institute of Cancer Neuroscience, Medical Frontier Innovation Research Center, The First Hospital of Lanzhou University, The First Clinical Medical College of Lanzhou University, Lanzhou, 730000 P. R. China

**Keywords:** Solute carrier transporters, Ferroptosis, Glucose, Lipid, Metal ion

## Abstract

Ferroptosis is a novel form of programmed cell death caused by damage to lipid membranes due to the accumulation of lipid peroxides in response to various stimuli, such as high levels of iron, oxidative stress, metabolic disturbance, etc. Sugar, lipid, amino acid, and iron metabolism are crucial in regulating ferroptosis. The solute carrier transporters (SLCs) family, known as the “metabolic gating” of cells, is responsible for transporting intracellular nutrients and metabolites. Recent studies have highlighted the significant role of SLCs family members in ferroptosis by controlling the transport of various nutrients. Here, we summarized the function and mechanism of SLCs in ferroptosis regulated by ion, metabolic control of nutrients, and multiple signaling pathways, with a focus on SLC–related transporters that primarily transport five significant components: glucose, amino acid, lipid, trace metal ion, and other ion. Furthermore, the potential clinical applications of targeting SLCs with ferroptosis inducers for various diseases, including tumors, are discussed. Overall, this paper delves into the novel roles of the SLCs family in ferroptosis, aiming to enhance our understanding of the regulatory mechanisms of ferroptosis and identify new therapeutic targets for clinical applications.

## Introduction

Ferroptosis, a unique form of regulated cell death, was first described by Stockwell and Dixon in 2012 [1]. This process is primarily triggered by an excess of iron and the abnormal metabolism of sugar, lipid, amino acid, etc, setting it apart from previously identified forms of cell death. Unlike apoptosis, necroptosis, and pyroptosis, which are considered active cell ‘suicides’, ferroptosis is characterized by a more passive cell ‘destruction’ [2, 3]. The hallmark of ferroptosis is the buildup of phospholipid peroxidation, particularly polyunsaturated fatty acids (PUFAs) and reactive oxygen species (ROS) [4–7]. The peroxidation of the plasma membrane triggered by excessive iron loading changes the fluidity and integrity of the plasma membrane, resulting in rupture of the mitochondrial outer membrane, reduction in mitochondrial size, reduction in number or loss of cristae, and ultimately loss of mitochondrial [8].Therefore, when ferroptosis occurs, the morphology of the cell undergoes necrotic changes, mainly characterized by cell membrane rupture, mitochondrial contraction, and increased density of the outer mitochondrial membrane. Dysregulation of ferroptosis has been implicated in various diseases, including neurological, cardiovascular, liver, gastrointestinal, lung, kidney, and pancreatic disorders [4, 9–11]. Ferroptosis may also provide an effective strategy to overcome tumor chemoresistance [12]. Various mechanisms of ferroptosis inhibition have been identified, with at least five cellular defense pathways known to counteract this process. These include the glutathione peroxidase 4 (GPX4)–reduced glutathione (GSH), the ferroptosis suppressor protein1(FSP1)–ubiquinol (CoQH2), the guanosine triphosphate (GTP) cyclohydrolase 1 (GCH1)–tetrahydrobiopterin (BH4)–LPs axis, the dihydroorotate dehydrogenase (DHODH)–CoQH2 regulatory system, and the recently discovered 7–DHC–DHCR7–cholesterol pathway [13, 14]. The identification of the 7–DHC–DHCR7–distal cholesterol pathway further suggests an important role of nutrient metabolism in regulating ferroptosis.

Indeed, ferroptosis is closely related to the metabolism of nutrients (including lipid, sugar, amino acid, and trace ion) [10, 15]. The transmembrane transport of these nutrients depends on Solute Carriers (SLCs) [16]. The SLCs superfamily is among the most important families of membrane transporters on the human cell membrane, and its members participate in material transport, energy transfer, nutrient metabolism, signal transduction, and other important physiological activities between cells [17]. This review summarizes the role and mechanism of nutrient metabolism regulation in ferroptosis, with a particular focus on the SLCs–related transporters that primarily transport five major components: glucose, amino acid, lipid, trace metal ion, and other ions. In addition, potential clinical applications of targeting SLCs in combination with ferroptosis inducers in various diseases are discussed.

## Nutrient metabolism and ferroptosis

Imbalance in nutrient metabolism, dysregulation of iron metabolism, and lipid peroxidation are major hallmarks of ferroptosis. Unlike other forms of regulated cell death, ferroptosis is primarily regulated by nutrient metabolic pathways. Metabolic and nutritional cues, including glucose, amino acid, fatty acid, energy transfer, endogenous antioxidants (such as coenzyme Q10, vitamin E, and di/tetrahydrobiopterin), and iron homeostasis can directly or indirectly regulate sensitivity to lipid peroxidation and ferroptosis [18–22].

**Fig. 1:**
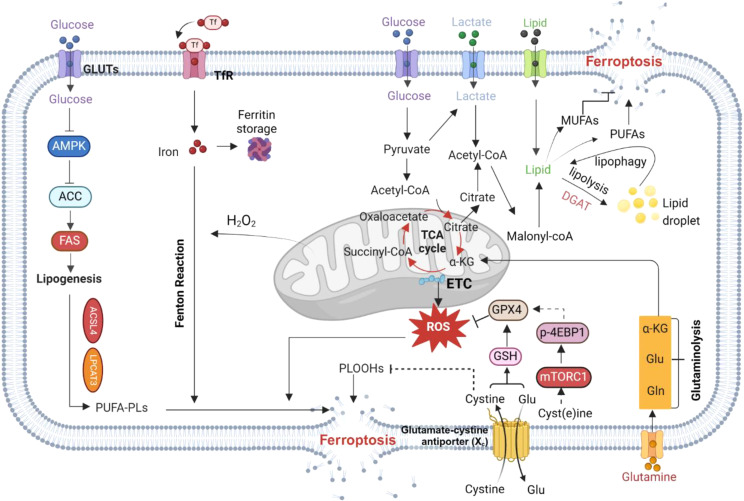
Regulation of iron and nutrient metabolism in ferroptosis. Changes in various signal transduction pathways affect ferroptosis sensitivity by regulating nutrient metabolism and homeostasis. Acetyl-CoA, which is produced from glycolysis, can be carboxylated to malonyl-CoA to synthesize lipids. AMPK signaling prevents ferroptosis, and glucose protects ferroptosis by antagonizing AMPK. Lipogenesis involves the generation of phospholipids containing polyunsaturated fatty acid chains (PUFA-PLs) mediated by long-chain ACSL4 and a variety of other enzymes, which are required for phospholipid peroxidation and ferroptosis. SLC7A11 transports cystine into the cell for GSH synthesis, and inhibition of SLC7A11 expression can induce ferroptosis. Cystine regulates GPX4 protein expression and ferroptosis through the mTORC1–4EBP pathway. Glutaminolysis plays a vital part in inducing ferroptosis under conditions of amino acid or cystine deprivation. After glutamine deamination, α-KG is generated and can directly enter the TCA, leading to accumulation of mitochondrial ROS and promoting ferroptosis. Levels of iron, another important factor in ferroptosis, can be increased by processes such as TFR-mediated iron import and autophagy-mediated ferritin degradation. Image created with BioRender. ETC, electron transport chain; Glu, glutamate; Gln, glutamine; PLOOH, phospholipid hydrogen peroxide

Glucose, lipid, and amino acid metabolism are crucial in regulating ferroptosis(Fig. 1). For instance, energy stress affects ferroptosis by activating AMP-activated protein kinase (AMPK), with consequent changes in various downstream signaling pathways. AMPK plays a key role in maintaining redox homeostasis and promoting cell survival during glucose starvation by regulating pathways related to fatty acid metabolism and antioxidant responses [18, 23]. On the other hand, AMPK activation promotes GPX4–dependent ferroptosis through the JAK2/STAT3/p53 signaling axis [24]. Glucose metabolism also serves as a major source of precursors for synthesis of macromolecules, including acetyl-CoA, which is needed for PUFA biosynthesis, and glycolysis intermediates used in the production of non-essential amino acid. Acetyl-CoA plays a dual role as a critical product of glycolysis and the starting point for de novo fatty acid synthesis [25]. PUFAs like arachidonoyl (AA) and adrenoyl (AdA) phospholipids, among others, which are catalyzed by various enzymes including long-chain acyl-CoA synthetase 4 (ACSL4), Lysophosphatidylcholine acyltransferase 3 (LPCAT3), and arachidonate lipoxygenases (ALOXs), can undergo oxidation leading to the formation of iron-dependent lethal lipid peroxides (LPO), and ultimately triggering ferroptosis [5, 26, 27]. By contrast, ACSL3 and stearoyl-CoA desaturase (SCD) facilitate the production of monounsaturated fatty acids (MUFAs) to inhibit ferroptosis [10, 28].

Usually, differentiated cells rely on mitochondrial oxidative phosphorylation for cell energy, while most cancer cells rely on aerobic glycolysis, a phenomenon called the “Warburg effect”. This leads to glycolysis becoming disconnected from the mitochondrial tricarboxylic acid (TCA) cycle and oxidative phosphorylation, hindering the conversion of glucose to acetyl-CoA into the TCA cycle [29]. To compensate for glucose deficiency, tumor cells show enhanced glutamine metabolism and replenish intermediate products of the TCA cycle as an energy supply. Glutaminolysis, a glutamine-dependent metabolic pathway, also has a vital role in inducing ferroptosis under conditions of amino acid or cystine deprivation. Glutaminolysis degrades glutamine to glutamate, which can be further converted to alpha-ketoglutarate (α-KG); this in turn can enter the TCA cycle and participate in mitochondrial ROS production, which is closely related to ferroptosis [30]. In addition, lipophagy and lipid hydrolysis also play important roles in regulating ferroptosis. For instance, RAS oncogene family 7 (RAB7)–mediated lipophagy and adipocyte triglyceride lipase (ATGL)–mediated lipolysis can increase free fatty acid production [31]. Triglyceride synthesis mediated by acylglycerol acyltransferase (DGAT1/2) and lipid droplet formation leads to accumulation of free fatty acids, thereby preventing peroxidation [32]. Lipolysis can generate PUFAs, which trigger lipid peroxidation and sensitize cells to ferroptosis [33, 34]. By contrast, lipases may produce MUFAs to decrease the levels of oxidized PUFAs in cell membranes, inhibiting lipid peroxidation and ferroptosis. Moreover, lipid droplets, acting as a ‘buffer zone’ for lipid storage and release, play a vital part in regulating ferroptosis sensitivity (Fig. 1).

## Iron and ferroptosis

In addition to phospholipid peroxidation of fatty acids, iron and ROS are required to drive ferroptosis. Iron enters the cell in the form of dietary Fe^3+^, which binds to transferrin (TF) on the cell membrane to form a TF– Fe^3+^ complex and is subsequently incorporated into the cell through TF receptor 1 (TFR1) – mediated endocytosis. It is then reduced to Fe^2+^ by six-transmembrane epithelial antigen of the prostate 3 (STEAP3), a key regulator of iron uptake, and stored in a labile iron pool (LIP) and ferritin [35, 36]. Cellular ROS, by contrast, are primarily generated by mitochondria, in which electron release from electron transport chain complexes I and III produces superoxide, which is then converted to H_2_O_2_ by superoxide dismutase (SOD) [37]. Then, H_2_O_2_ can react with free Fe^2+^ in the cell by the Fenton reaction to generate hydroxyl radicals, which react directly with PUFAs in the cellular or plasma membrane, leading to the formation of phospholipid hydroperoxides (PLOOHs) and ultimately initiating ferroptosis [37, 38] (Fig. 1). However, this process can be regulated by various methods, including oxidization of intracellular Fe^2+^ to Fe^3+^ by iron transporter ferroportin (FPN1; also known as SLC40A1) [39] and control of iron homeostasis by heat shock protein family B member 1 [40] and nuclear receptor coactivator 4 (NCOA4) [41] to prevent the accumulation of high concentrations of intercellular iron that could induce ferroptosis. Research has also shown that dynamic changes in the iron redox state are crucial for determining sensitivity of cell to ferroptosis [15, 42]. In summary, the regulation of ferroptosis is affected by imbalance in nutrients and intracellular iron levels, which are mediated by SLC transporters. The potential roles of SLCs in ferroptosis have been verified and paid more attention.

## SLCs and ferroptosis

SLCs are a family of transporters that regulate the movement of molecules across cell membranes and have crucial roles in controlling metabolic processes within cells. The human SLCs transporter superfamily comprises 65 subfamilies, totaling 458 members [43]. As per the guidelines of the Human Genome Organization (HUGO) Gene Nomenclature Committee (HGNC), SLCs are designated with the root symbol SLC, followed by a numerical value (e.g., SLC16, representing solute carrier family 16), the letter A (used as a separator), and the specific transporter number (e.g., SLC16A1). The key criterion for classifying SLCs family members is that each member should have 20–50% sequence similarity with at least one other member [44, 45]. SLCs generally consist of 1–16 transmembrane domains, with a pseudosymmetry in their core transmembrane domain. These transmembrane proteins are commonly found on the surfaces of various organelles, such as the cell membrane, endoplasmic reticulum, mitochondria, lysosomes, and peroxisomes. They facilitate the transport of a wide range of substrates, including sugar, amino acid, lipid, vitamin, and metal ion (Table 1), and play crucial parts in numerous biological processes, including regulation of cell signaling and organizing cellular organelles [43, 46]. The SLC2 and SLC5 families facilitate the transport of glucose and fructose; the SLC1, SLC3, SLC6, SLC7, SLC36, SLC38, and SLC43 families drive amino acid transport; the SLC19, SLC46, and SLC23 families are responsible for the transport of vitamins; the SLC11, SLC30, SLC31, SLC39, SLC40, SLC41, and SLC49 families regulate the transport of trace metals; and SLC10 and SLC22 transport bile acids, steroid hormones, and similar compounds. The main fatty acid transporters are the fatty acid transporter family (FATPs), fatty acid translocases (FAT/CD36), and plasma membrane fatty acid binding proteins (FABPs). FATPs, also known as SLC27 family proteins, belong to the SLC superfamily [43, 47, 48].

**Table 1:** Information on SLCs associated with ferroptosis

Category	Gene	Alisas	Predominant substrates	The relationship with ferroptosis
Glucose metabolism-associated SLCs	SLC16A1	HHF7, MCT, MCT1, MCT1D	Monocarboxcylates	Inhibitor
	SLC2A1	CSE, DYT17, DYT18, DYT9, EIG12, GLUT, GLUT-1, GLUT1, GLUT1DS, HTLVR, PED, SDCHCN	Glucose, galactose, mannose, glucosamine	Inducer
Lipid metabolism-associated SLCs	SLC47A1	MATE1	Lipid, metformin, memantine, MPP, tetraethylammonium	Inhibitor
	SLC27A4	IPS; FATP4; ACSVL4	Translocation of long-chain fatty acid	Inhibitor
	SLC27A5	BAL; ACSB; BACS; FATP5; ACSVL6; FACVL3; FATP-5; VLACSR; VLCSH2; VLCS-H2	Lipid	Inducer
	SLC27A2	ACSVL1, FACVL1, FATP2, HsT17226, VLACS, VLCS, hFACVL1	Lipid	Inducer
Amino acid metabolism-associated SLCs	SLC38A9	SNAT9; URLC11	Alkaline amino acid	Inhibitor
	SLC38A2	ATA2, PRO1068, SAT2, SNAT2	Neutral amino acid	Inhibitor
	SLC25A22	GC1; DEE3; GC-1; EIEE3; NET44	Glutamate	Inhibitor
	SLC7A11	CCBR1, xCT	Amino acid	Inhibitor
	SLC25A39	CGI-69, CGI69	Mediated glutathione homeostasis	Inhibitor
	SLC3A2	4F2, 4F2HC, 4T2HC, CD98, CD98HC, MDU1, NACAE	Neutral, dibasic and large amino acid	Inhibitor
	SLC1A5	R16; AAAT; ATBO; M7V1; RDRC; ASCT2; M7VS1	Glutamine	Inducer
	SLC43A2	LAT4	Methionine and phenylalanine	Inducer
	SLC6A14	BMIQ11	Neutral and cationic amino acid	Inducer
Trace metal metabolism-associated SLCs	SLC40A1	FPN; FPN1; HFE4; MTP1; IREG1; MST079; MSTP079; SLC11A3	Iron exporter	Inhibitor
	SLC39A7	KE4; AGM9; HKE4; ZIP7; RING5; H2-KE4; D6S115E; D6S2244E	Zinc	Inducer
	SLC39A14	HCIN; NET34; ZIP14; cig19; HMNDYT2; LZT-Hs4	Iron importer	Inducer
	TFRC	CD71, IMD46, T9, TFR, TFR1, TR, TRFR, p90	Iron importer	Inducer
	SLC25A28	MFRN2; MRS4L; MRS3/4; NPD016	Ferrous iron	Inducer
	SLC11A2	AHMIO1, DCT1, DMT1, NRAMP2	Metals	Inducer
Other ions-transporting SLCs	UCP2	UCPH; BMIQ4; SLC25A8	Mitochondrial proton anion ( Cl^−^, Br^−^, and NO3 ^−^)	Inhibitor
	SLC25A10	DIC; MTDPS19	Dicarboxylate carrier	Inhibitor
	SLC25A11	OGC, PGL6, SLC20A4	Oxoglutarate	Inhibitor

Given the relationship between nutrient balance and ferroptosis, the roles of SLCs as transporters of the main three major nutrients (sugar, lipid and amino acid) would suggest direct or indirect involvement in the regulation of ferroptosis. Indeed, various SLCs are known to play important parts in various mechanisms of ferroptosis via lipid and carbon metabolism (Fig. 1). Specifically, SLC7A11, a member of the solute carrier family, serves as a key regulator of the canonical ferroptosis inhibition system [49]. However, despite an increasing research focus on the involvement of SLC family members in ferroptosis regulation in recent years, there has not yet been a comprehensive summary of their roles in this regard.

Therefore, in this review, we aim to outline the regulatory relationships between SLCs family members and ferroptosis (Table 1) to shed light on the potential contributions of SLCs transmembrane transporter families to this process. We divide SLCs into five categories according to their substrates: (1) SLCs associated with glucose metabolism; (2) SLCs related to amino acid metabolism; (3) SLCs associated with lipid metabolism; (4) trace-metal-related SLCs; and (5) SLCs associated with other ions. By delineating the main signaling pathways and the key genes and proteins involved in the regulatory roles of SLCs in ferroptosis (Table 2), we provide a comprehensive overview of the intricate connections between SLCs and ferroptosis.

**Table 2:** Information of crucial signaling pathways involved in SLCs associated with ferroptosis

Category	Gene Name	The involved signaling pathways	The involved key genes /proteins	Refs
Glucose metabolism-associated SLCs	SLC16A1(MCT1)	AMPK-SREBP1-SCD1 signaling pathway	AMPK, SREBP1, SCD1	[61]
	SLC2A1	HIF1αsignaling pathways	HIF-α	[53]
Lipid metabolism-associated SLCs	SLC47A1	PPARα signaling pathway	PPARA, PUFA- ce	[102]
	SLC27A4	MUFAs uptake	MUFAs	[91]
	SLC27A5	NRF2-GSH signaling pathway	NRF2,GSH	[92]
	SLC27A2	PUFA-PLs synthesis	AA, PUFA-PLs	[93]
Amino acid metabolism-associated SLCs	SLC38A9	mTORC1 signaling pathway	mTORC1, SLC7A11, GPX4	[80]
	SLC38A2			[76]
	SLC25A22	AMPK-SREBP1-SCD1 signaling pathway	AMPK, SCD1	[81]
	SLC25A39	GSH-GPX4 signaling pathway	GPX4,GSH	[84]
	SLC7A11	GSH-GPX4 or mTORC1 signaling pathway	GPX4,GSH, p70S6K, mTORC1,4EBPs	[49, 68, 138]
	SLC3A2			[78, 79]
	SLC43A2	NFκB signaling pathway	IKKα/β, p65, GPX4	[80, 88]
	SLC6A14	C/EBPβ-PAK6 signaling pathway	PAK6, C/EBPβ	[87]
Trace metal metabolism-associated SLCs	SLC40A1	Iron metabolism	Iron, ROS, H_2_O_2_	[113]
	SLC39A7(ZIP7)	ER stress-HERPUD1 signaling pathway	HERPUD1, ATF3	[115]
	SLC11A2(DMT1)	SIRT3/IDH2 signaling pathway	SIRT3, IDH2, GSH	[108, 109]
	SLC39A14(ZIP14)	Hippo signaling pathway	YAP	[107]
	SLC25A28	BRD-P53 signaling pathway	BRD, P53	[112]
Other ion-transporting SLCs	UCP2	UCP2/SIRT3/PGC1α signaling pathway	UCP2,SIRT3,PGC1α	[65]
	SLC25A10	GSH-GPX4 signaling pathway	GPX4,GSH	[120]
	SLC25A11			

### Sugar metabolism-associated SLCs and ferroptosis

Glucose, fructose, and galactose are the three simple sugar that are crucial in nutritional signaling. Glucose, a vital nutrient for cellular energy production, is essential for ferroptosis. Under glucose-deficient conditions, the intracellular AMP/ATP ratio increases, leading to activation of AMPK, a cellular metabolic and energy sensor, which in turn phosphorylates and inhibits its downstream substrate acetyl-CoA carboxylase 1 (ACC1). This inhibits the synthesis of fatty acids, thereby slowing the accumulation of lipid oxides and the occurrence of ferroptosis [23]. Energy stress also triggers inhibition of ferroptosis mediated by activation of AMPK, whereas inactivation of AMPK sensitizes cells to ferroptosis [18, 23]. The role of glucose in the regulation of ferroptosis was further confirmed by experiments showing that glucose deprivation could inhibit the effects of erastin and RSL3, which induce ferroptosis via mimicking the effects of cystine starvation and preventing system Xc^−^-mediated cystine uptake.

The SLC2 family has a key role in transporting carbohydrates such as monosaccharides and polyols. For instance, SLC2A1 transports not only glucose but also mannose, galactose, and glucosamine, and there have been extensive studies of its involvement in tumor progression [43, 50, 51]. It is also related to ferroptosis, as it promotes glycolysis, accelerates pyruvate oxidation, provides intermediate metabolites for the TCA, and stimulates fatty acid synthesis, ultimately leading to lipid-peroxidation-induced ferroptosis [52]. In addition, SLC2A1 can be activated by capsiate ester, a metabolite of intestinal flora, which inhibits HIF-1α and reduces the progression of ferroptosis-associated osteoarthritis [53].

The SLC5 family, also known as SGLTs, function as transporter of monosaccharides including glucose, galactose, and fructose. SLC5A1 (SGLT1), the best-known member of this family, is responsible for active glucose and galactose absorption in the intestine, as well as glucose reabsorption in the kidney [43]. Unlike glucose, fructose can be converted to fructose 1,6-bisphosphate, allowing it to enter the glycolysis process. Fructose can reprogram cellular metabolic pathways, promoting glutaminolysis and oxidative metabolism. Glutaminolysis plays a crucial part in ferroptosis triggered by cystine starvation, and studies have shown that elevated fructose levels can lead to ferroptosis in glomerular podocytes [29, 30, 54, 55]. Galactose is also associated with ferroptosis: injection of D-galactose downregulates expression of GPX4 mRNA [48] in mouse livers and induces ferroptosis in mouse femoral osteoblasts [56], and ferroptosis has been implicated in D-galactose-induced hearing loss in mice [57]. Given the regulatory roles of fructose and galactose in ferroptosis, it is crucial to further investigate the relationship between the SGLT family and ferroptosis in future studies.

According to the Warburg effect, tumor cells metabolize large amounts of glucose, leading to the accumulation of lactic acid through aerobic glycolysis. Lactic acid, in a pH-dependent manner, can enhance cellular resistance to ferroptosis [58]. The transportation of lactic acid in tumor cells is facilitated by solute transporters such as SLC16A1 (monocarboxylic acid transporter 1, MCT1), SLC16A7 (monocarboxylic acid transporter 2, MCT2), and SLC16A3 (monocarboxylic acid transporter 4, MCT4), which play a role in maintaining the balance of intracellular energy and pH [59, 60]. MCT1–mediated lactate uptake promotes ATP production and inactivates AMPK in hepatocellular carcinoma, resulting in upregulation of sterol regulatory element binding protein 1 (SREBP1) and stearoyl-CoA desaturase 1 (SCD1), thereby promoting production of the MUFAs against ferroptosis [61]. Depletion of MCT4 can inhibit lactic acid and acidify the intracellular milieu, both of which can improve the ferrocene-catalyzed lipid peroxidation and induce ferroptosis [62].

**Fig. 2:**
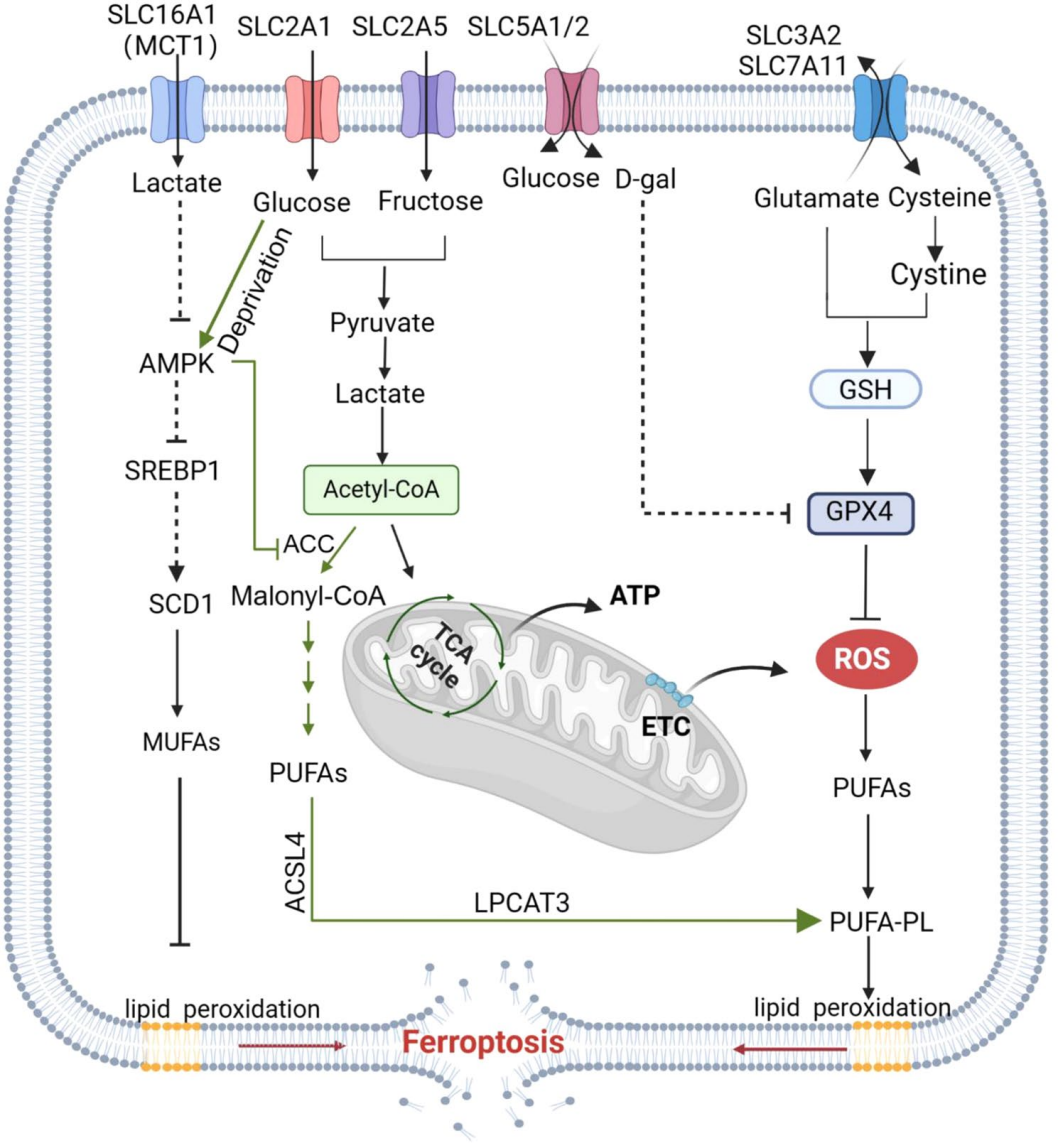
Regulation of sugar-associated SLC transporters in ferroptosis. Glucose and fructose are transported into cells through SLC2A1/5 to participate in the TCA cycle, which in turn promotes ROS accumulation, lipid peroxidation and phosphorylation, and ferroptosis. AMPK signaling prevents ferroptosis, and glucose promotes ferroptosis by antagonizing AMPK. Glycolysis product lactic acid can also enter the cell through SLC16A1 and inhibit synthesis of MUFAs and thus ferroptosis by inhibiting the AMPK–SCD1 signaling pathway. Galactose can enter the cell through SLC5A1/2 and inhibit ferroptosis by inhibiting GPX4 activity. Image created with BioRender

In conclusion, glucose, fructose, galactose, and lactic acid are all closely associated with ferroptosis (Fig. 2), and the detailed roles of SLC family members as transporters of these nutrients in ferroptosis require further exploration and characterization.

### Amino acid-associated SLCs and ferroptosis

Emerging evidence indicates the significant involvement of amino acid metabolism in ferroptotic cell death. For instance, glutaminolysis, the TCA cycle, and electron transport chains (ETC) have been identified as crucial components in cystine starvation-induced ferroptosis within cancer cell metabolism. ROS accumulation, lipid peroxidation, and ferroptosis are not induced in the absence of glutamine, inhibition of glutamate metabolism, cystine starvation, or inhibition of cystine entry into the cytoplasm [30, 63].

Glutamine, in particular, is a key extracellular regulator of ferroptosis that can be degraded by glutaminolysis, serve as an energy source for the TCA cycle, and promote lipid biosynthesis. Glutamine is transported into cells via amino acid transporters SLC1A5, SLC38A1, and SLC38A2. Inside mitochondria, glutaminase converts glutamine to glutamate which has an important role in GSH biosynthesis and contributes to the GSH–GPX4 defense axis in ferroptosis. This mitochondrial glutamate is further converted by glutamate dehydrogenase 1 (GLUD1) into α-KG, a crucial intermediate metabolite that modulates the overall rate of the TCA cycle and also has a role in fatty acid biosynthesis and ATP generation. In addition, mitochondrial glutamate can be exported from the mitochondria to the cytosol through transporters SLC25A18 and SLC25A22, contributing to the biosynthesis of GSH [64]. Mitochondrial glutamine-derived aspartate is also transported into the cytoplasm, in a process facilitated by SLC25A8 (UCP2) [65]. Sirtuin 3 (SIRT3), is the primary NAD^+^-dependent deacetylase in mitochondria. Its main function is to protect mitochondria from damage. SIRT3 regulates mitochondrial production by modulating downstream antioxidant PGC1α, alleviating cellular oxidative damage, and enhancing mitochondrial performance; thus, it has a role in various diseases, including cancer. However, the UCP2/SIRT3/PGC1α pathway can protect cells from oxidative damage caused by increased mitochondrial ROS levels, which are known to promote ferroptosis; in this way, mitochondrial homeostasis is maintained and cells are enabled to resist ferroptosis. It has also recently been reported that matrine can suppress ferroptosis hyperactivation and reduce ROS production by activating the UCP2/SIRT3/PGC1α pathway in the lung [66].

Cancer cells have high levels of oxidative stress owing to neo-biosynthesis and protein catabolism, which poses a challenge for the cells in terms of meeting their antioxidant defense needs. Most cancers primarily depend on import of extracellular cystine by system Xc^−^, which comprises SLC7A11and SLC3A2 subunits, for antioxidant defense [49]. Inside the cell, the imported cystine is reduced to cysteine, which is then used to synthesize GSH, the main intracellular antioxidant. GSH is an essential cofactor of GPX4, which can convert GSH to oxidized glutathione, while reducing lipid peroxides, thereby alleviating oxidative stress damage and inhibiting ferroptosis [67]. In 2012, Dixon and Stockwell discovered that ferroptosis could be induced by inhibition of SLC7A11- mediated cystine uptake by erastin or depletion of cystine [1], and inhibition of SLC7A11 activity through cystine starvation, gene silencing, or use of inhibitors has been shown to induce ferroptosis in various cell types, including cancer cells. Recent studies have also shown that KRAS or ATF4 could promote SLC7A11 transcription, resulting in increased SLC7A11-mediated cystine uptake and GSH synthesis, helping to maintain intracellular redox balance and confer resistance to ferroptosis [1, 49, 68–70]. Expression of SLC7A11 is mediated by ATF4 and nuclear factor erythrogen 2 related factor 2 (NRF2), and p53 and ATF3 (p53 independent) act as key repressors of its transcription. SLC3A2 forms heterodimers not only with SLC7A11 but also with SLC7A5-8 and SLC7A10, facilitating the transport of cystine and various l-type amino acid [71]. Moreover, SLC1A1, an essential glutamate transporter that facilitates cystine uptake, is involved in the mechanism by which hypoxic transcription factor HIF-1α increases resistance to ferroptosis; HIF-1α both induces lactic acid in a pH-dependent manner and upregulates the expression of SLC1A1 [58]. SLC1A5, another member of the SLC1A family, has been implicated in the regulation of ferroptosis and immune response in gliomas and could serve as a prognostic biomarker for gliomas and a potential therapeutic target [72].

Another crucial pathway, mTORC1, a key player in nutrient sensing, has been found to inhibit ferroptosis by stimulating protein synthesis independently of the GPX4–GSH axis (Fig. 1). The activity of mTORC1 is modulated by multiple upstream stimuli, including amino acid such as leucine and arginine, glucose, and growth factors. Upon activation, mTORC1 phosphorylates its downstream effectors, which include p70S6 kinase and eukaryotic initiation factor 4E (eIF4E)-binding proteins (4EBPs) to promote protein biosynthesis [73, 74]. SLC38A1, a member of the SLC38 family of glutamine transporters that facilitate glutamine influx into cells, plays a critical part in the regulation of glutamine uptake and metabolism in lipid peroxidation [75], and both SLC38A1 and SLC1A5 co-transport polarized glutamine and sodium, with the intracellular glutamine being exchanged for extracellular leucine via CD98 (SLC7A5 and SLC3A2 complexes), thereby promoting mTORC1 activation [16]. SLC38A2, which has been identified as a novel osmoresponsive neutral amino acid transporter, also protects renal medullary collecting duct cells from hyperosmotic-stress-induced ferroptosis by activating the mTORC1 signaling pathway [76]. SLC7A11-mediated cystine uptake, as well as promoting GSH synthesis, also promotes GPX4 protein synthesis, partly through the Rag–mTORC1–4EBP signaling axis [77](Fig. 1). SLC3A2 deficiency promotes ferroptosis by upregulating the expression of the mTOR and p70S6K in laryngeal carcinoma [78], whereas targeting of the SLC3A2 subunit of system Xc^−^ is essential for N^6^-methyladenosine reader YTHDC2 (YT521-B homolog 2) to function as an endogenous ferroptosis inducer in lung adenocarcinoma [79]. Arginine transporter SLC38A9 can also activate the mTOR signaling pathway to regulate ferroptosis sensitivity by mediating SLC7A11/GPX4 expression and ferritinophagy [80]. SLC25A22 has been identified as a novel driver of ferroptosis resistance in pancreatic ductal adenocarcinoma cells that exerts its effects through production of GSH and MUFAs [81]. In addition, loss of SLC25A39 can reduce mitochondrial GSH import and abundance without affecting cellular GSH levels and dysregulate the protein activity and stability of iron-sulfur clusters in mammalian cells [82]. Notably, insufficient maintenance of iron-sulfur clusters can activate the iron starvation response, which works in conjunction with glutathione biosynthesis to initiate ferroptosis [83].

Glycine is an important amino acid in ferroptosis-related mechanisms. Exogenous glycine has been shown to increase GSH levels and expression of antioxidant-related genes, such as GPX4 and SLC25A39, thereby regulating ROS-induced lipid metabolism to protect against ferroptosis. Exogenous glycine has been shown to increase GSH levels and expression of antioxidant-related genes, such as GPX4 and SLC25A39, thereby regulating ROS-induced lipid metabolism to protect against ferroptosis. Additionally, glycine plays a crucial role in swine oocyte maturation and later development [84]. SLC6A14, which imports all neutral amino acid and the two cationic acid lysine and arginine into the cytoplasm, is upregulated in various colonic diseases, including ulcerative colitis [85]. It is also associated with P21 (RAC1)-activated kinase 6 (PAK6), inhibition of which has been shown to sensitize therapy-resistant cells to tyrosine kinase inhibitors in chronic myeloid leukemia by disrupting the RAS/MAPK pathway and mitochondrial activity [86]. SLC6A14 negatively regulates PAK6 in a manner dependent on CCAAT enhancer binding protein beta (C/EBPβ), promoting epithelial cell ferroptosis in ulcerative colitis via the C/EBPβ–PAK6 axis [87].

Methionine metabolism also plays an important role in the regulation of ferroptosis, and methionine is required for ferroptosis induction via blockade of cystine uptake. Intermittent dietary methionine deprivation has been shown to promote tumor cell ferroptosis, and combining this deprivation with system Xc^−^ inhibitors and PD-1 blockade enhances antitumor efficacy. Methionine/cystine restriction (MCR) and inhibition of SLC43A2, a methionine transporter, suppress tumor progression of esophageal squamous cell carcinoma by diminishing phosphorylation of IKKα/β and p65 [88]. Mechanistically, SLC43A2-mediated intake of methionine activates the NFκB signaling pathway, resulting in elevated mRNA expression of SLC43A2 and GPX4, whereas elevated methionine levels also increase levels of GSH. The resulting positive feedback loop between SLC43A2 and NFκB signaling reduces ferroptosis and promotes tumor progression [88].

**Fig. 3:**
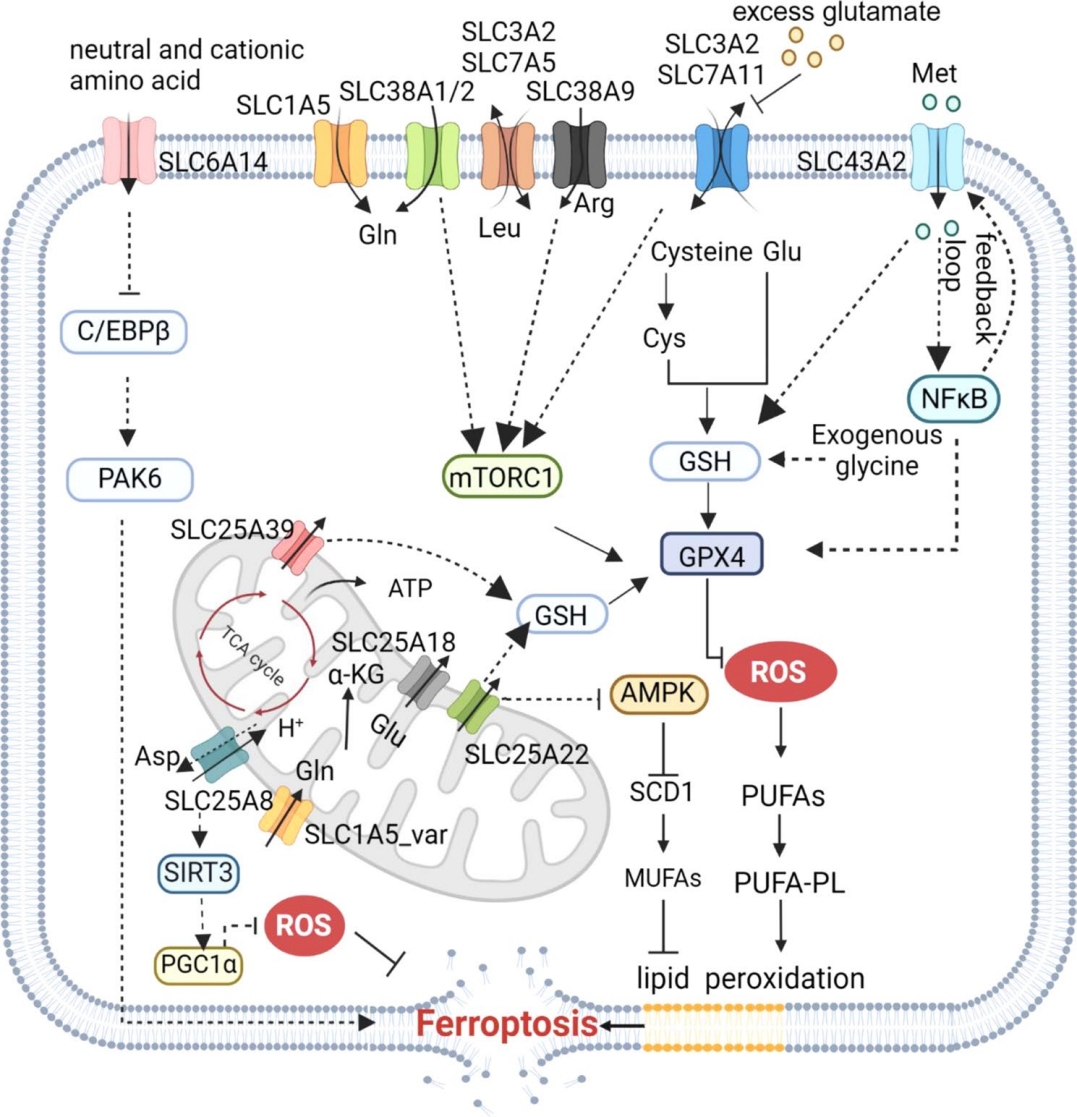
Regulation of amino acid-related SLC transporters in ferroptosis. SLC7A11-mediated cystine uptake not only promotes GSH synthesis but also inhibits ferroptosis in part through the mTORC1–4EBP signaling axis. SLC38A1 or SLC1A5 co-transports polarized glutamine, with intracellular glutamine being exchanged for extracellular leucine via CD98 (SLC7A5 and SLC3A2 complexes), thereby promoting mTORC1 activation. Arginine transporter SLC38A9 and neutral amino acid transporter SLC38A2 can also regulate ferroptosis sensitivity by mediating activation of the mTOR signaling pathway. SLC25A22, SLC25A39, and SLC43A2 can increase GSH levels to inhibit ferroptosis. In addition, SLC25A22 leads to production of anti-ferroptosis MUFAs and inhibits ferroptosis in an AMPK-dependent manner. SLC25A8(UCP2)/SIRT3/PGC1α can reduce ROS production to inhibit ferroptosis. SLC6A14, which negatively regulates PAK6 expression in a C/EBPβ-dependent manner, promotes ferroptosis. Image created with BioRender

These findings collectively demonstrate that amino acid have crucial roles in the regulation of ferroptosis (Fig. 3); as the synthesis, degradation, and transport of amino acid are dependent on specific amino acid transporters, the roles of SLCs are also crucial to this process. Notably, glutamate and glutamine act as key positive regulators of cell ferroptosis; the interplay between glutamate and cystine relies on system Xc^−^, the function of which can be hindered by elevated levels of glutamate, leading to initiation of ferroptosis [89]. In addition, breakdown of glutamine can supply the TCA with raw materials and support various biosynthetic processes such as lipid biosynthesis. Thus, the role of amino acid transporters is integral to the regulation of amino acid metabolism and consequently the control of ferroptosis.

### Lipid metabolism-associated SLCs and ferroptosis

**Fig. 4:**
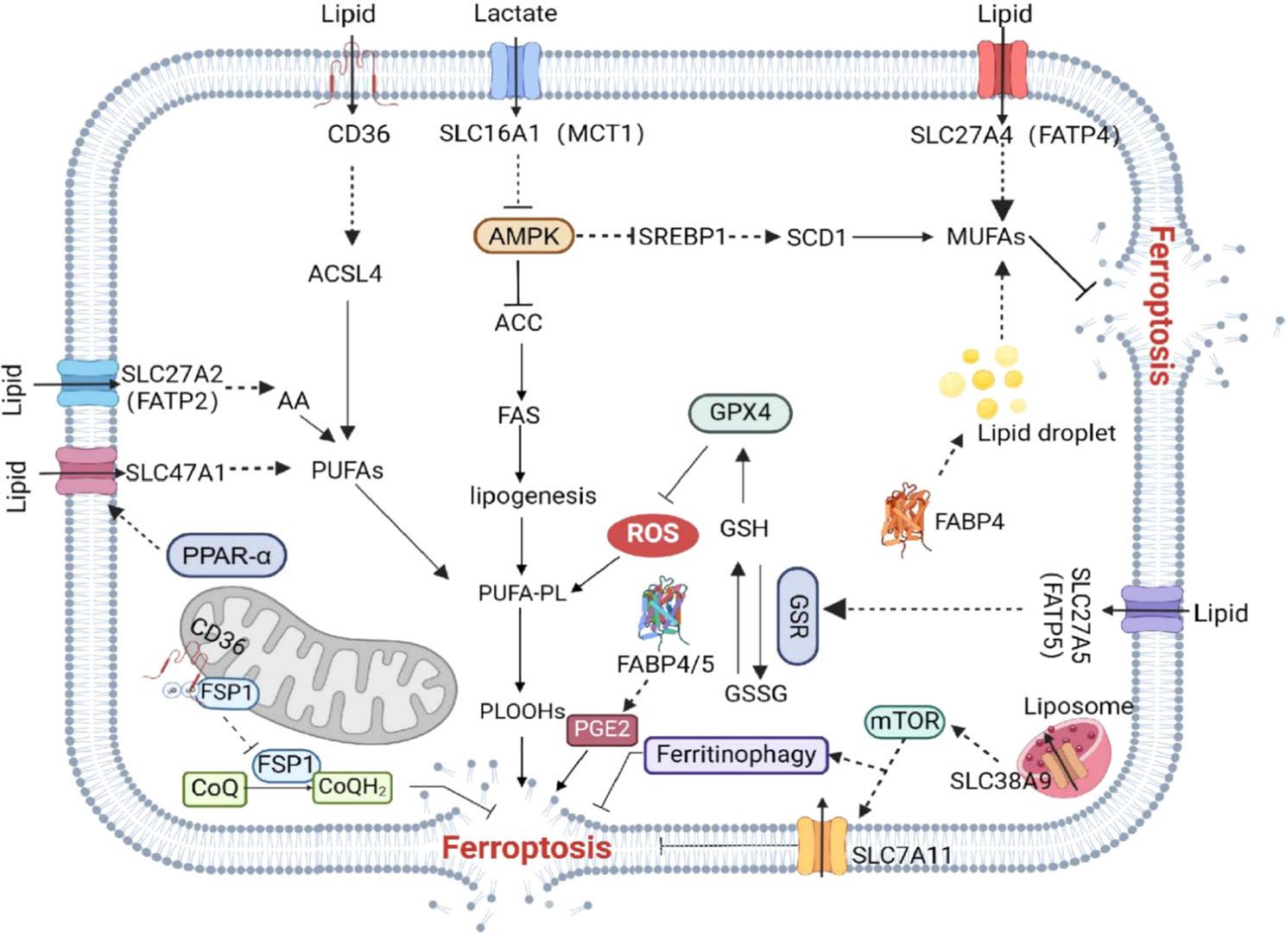
Regulation of lipid-related SLC transporters in ferroptosis. The proteins involved in the transport of fatty acids include CD36, FABPs, and FATPs. Specifically, an increase in SLC27A2 (FATP2) levels triggers ferroptosis by modulating the accumulation of arachidonic acid as PUFA-PLs. SLC27A4 (FATP4) inhibits ferroptosis by significantly enhancing the selective uptake of MUFAs. SLC27A5 (FATP5) promotes sorafenib-induced ferroptosis by upregulating the expression of glutathione reductase (GSR) in an NRF2-dependent manner, disrupting the balance of GSH homeostasis. FABP4 and SCD1 can co-stimulate the formation of lipid droplets (LD), promote synthesis of MUFAs, and inhibit ferroptosis. FABP4/5 deficiency in macrophages results in reduced production of prostaglandin E2 (PGE2), which is also an inducer of ferroptosis. CD36 specifically binds to FSP1 and mediates ubiquitination, leading to FSP1 degradation and ferroptosis. In addition, PPARA-dependent SLC47A1 expression prevents ferroptosis by inhibiting the production of PUFA esterified cholesterol esters (PUFA-CE) for lipid peroxidation. SLC38A9 regulates the sensitivity of ferroptosis by sensing and transducing intracellular cholesterol, activating the mTOR signaling pathway, and upregulating SLC7A11/GPX4 expression and ferroautophagy.Image created with BioRender

Ferroptosis is characterized by accumulation of lipid peroxidation on cellular membranes, which occurs in an iron-dependent manner, and the sensitivity of cells to this process is closely linked to various metabolic and signaling pathways. Similar to other types of regulated necrotic cell death, ferroptosis involves membrane damage; this has been proposed to be caused by iron overload, leading to alterations in membrane fluidity and permeability [50, 90]. Cellular lipids and lipid metabolism are crucial in regulating ferroptosis(Fig. 4).

Transporters involved in transporting fatty acids include CD36, FABPs, and FATPs. Members of the FATP or SLC24 family function as long-chain FATP and acyl-CoA synthetases with long-chain acyl-CoA synthetase activity and participate in a variety of metabolic processes, including fatty acid uptake and fatty acid signaling pathways. They are mainly localized within cells and the cellular membrane and have key roles in long-chain fatty acid transport. Different members of the SLC27 family have been reported to have contrasting effects on regulation of ferroptosis in liver cancer. For example, overexpression of SLC27A4, also known as FATP4, inhibits ferroptosis in hepatoma cells by significantly enhancing selective uptake of MUFAs [91]. On the other hand, SLC27A5 (also known as FATP5), which serves as a suppressor in sorafenib-resistant hepatocellular carcinoma cells, markedly promotes sorafenib-induced ferroptosis by upregulating expression of glutathione reductase in an NRF2-dependent manner, disrupting the balance of GSH homeostasis [92]. SLC27A2 (also referred to as FATP2), a key regulator of the immunosuppressive function in polymorphonuclear myeloid-derived suppressor cells, plays a crucial part in inducing ferroptosis; an increase in FATP2 in PMN-MDSCs in these cells triggers ferroptosis by modulating the accumulation of arachidonic acid in the form of polyunsaturated fatty acid phospholipids [93].

FABPs were originally identified as intracellular molecules that affect lipid flux, metabolism, and signaling in cells; they are also are important mediators of metabolism that support systemic homeostatic networks of immunometabolism by facilitating signaling within cells and communication between organs. FABP3, a cytosolic carrier of PUFAs, has been shown found to decrease membrane fluidity and induce endoplasmic reticulum stress by altering the lipid composition of the plasma membrane in aged muscles. This indicates a potential correlation between FABP3 and ferroptosis. In addition, FABP4 has been identified as a downstream target of the peroxisome proliferation activation receptor γ (PPARγ) signaling pathway in macrophages and human monocytes; and impaired PPARγ signaling in neurons leads to iron accumulation, exacerbating neuronal ferroptosis [90, 94]. In the context of recurrent human breast cancer, FABP4 and SCD1 within the tumor microenvironment work together to stimulate the formation of lipid droplets and facilitate the synthesis of MUFAs, providing protection against ferroptosis triggered by oxidative stress [95]. FABP4/5 deficiency in macrophages can result in decreased production of prostaglandin E2 (PGE2), which is also an inducer of ferroptosis [96, 97]. There is evidence that FABPs can interact directly with CD36 [98]. CD36, also known as scavenger receptor, has crucial roles in lipid metabolism and insulin resistance and has recently been reported to also regulate ferroptosis. As well as inducing lipid oxidation and ferroptosis via mediation of fatty acid uptake of tumor-infiltrating CD8^+^ T cells in the tumor microenvironment [99], its high expression triggers pericyte mitosis-dependent ferroptosis, resulting in destruction of the blood–brain barrier [100]. Moreover, in adult mice subjected to acute kidney injury, CD36 specifically binds to FSP1 and mediates K16- and K24-linked ubiquitination, leading to FSP1 degradation and ferroptosis [101].

The lipid flippase SLC47A1 is identified as a regulator of lipid remodeling and survival to suppress metabolic vulnerability to ferroptosis among 49 phospholipid scramblases, flippases, and floppases. Mechanistically, SLC47A1 expression dependent on transcription factor PPARA prevents ferroptosis by inhibiting the production of PUFA esterified cholesterol esters for lipid peroxidation [102]. The SLC25A20 transporter, also known as carnitine acyl-carnitine carrier, is the main means of entry of fatty acids into mitochondria; it exchanges free carnitine for the fatty acid acyl-carnitine, followed by fatty acid oxidation in the inner mitochondrial membrane [103]. Although there is no direct evidence of SLC25A20 in regulating ferroptosis, its role in mitochondrial fat acid oxidation cannot be ignored. Similarly, although the primary function of lysosomal transmembrane-resident protein SLC38A9 is amino acid transport, particularly that of arginine, it has been reported to also sense and transduce intracellular cholesterol, thereby regulating the ferroptosis sensitivity of hematopoietic stem cells by activating the mTOR signaling pathway, upregulating SLC7A11/GPX4 expression, and promoting ferritinophagy [80].

Given the roles of fatty acids in ferroptosis discussed above, it follows that the various mechanisms by which fatty acids enter a cell can modulate its ferroptosis sensitivity. Unesterified fatty acids can be taken into the cell by FATPs. These mechanisms include uptake of unesterified fatty acids into the cell by FATPs, and removal of unesterified fatty acids from the extracellular environment and binding of lipoproteins by CD36 to facilitate cholesterol transport. Therefore, dysfunction of FATPs can affect lipid metabolism, with consequent impacts on the regulation of ferroptosis through various signaling pathways (Fig. 4).

### Trace metal-associated SLCs and ferroptosis

**Fig. 5:**
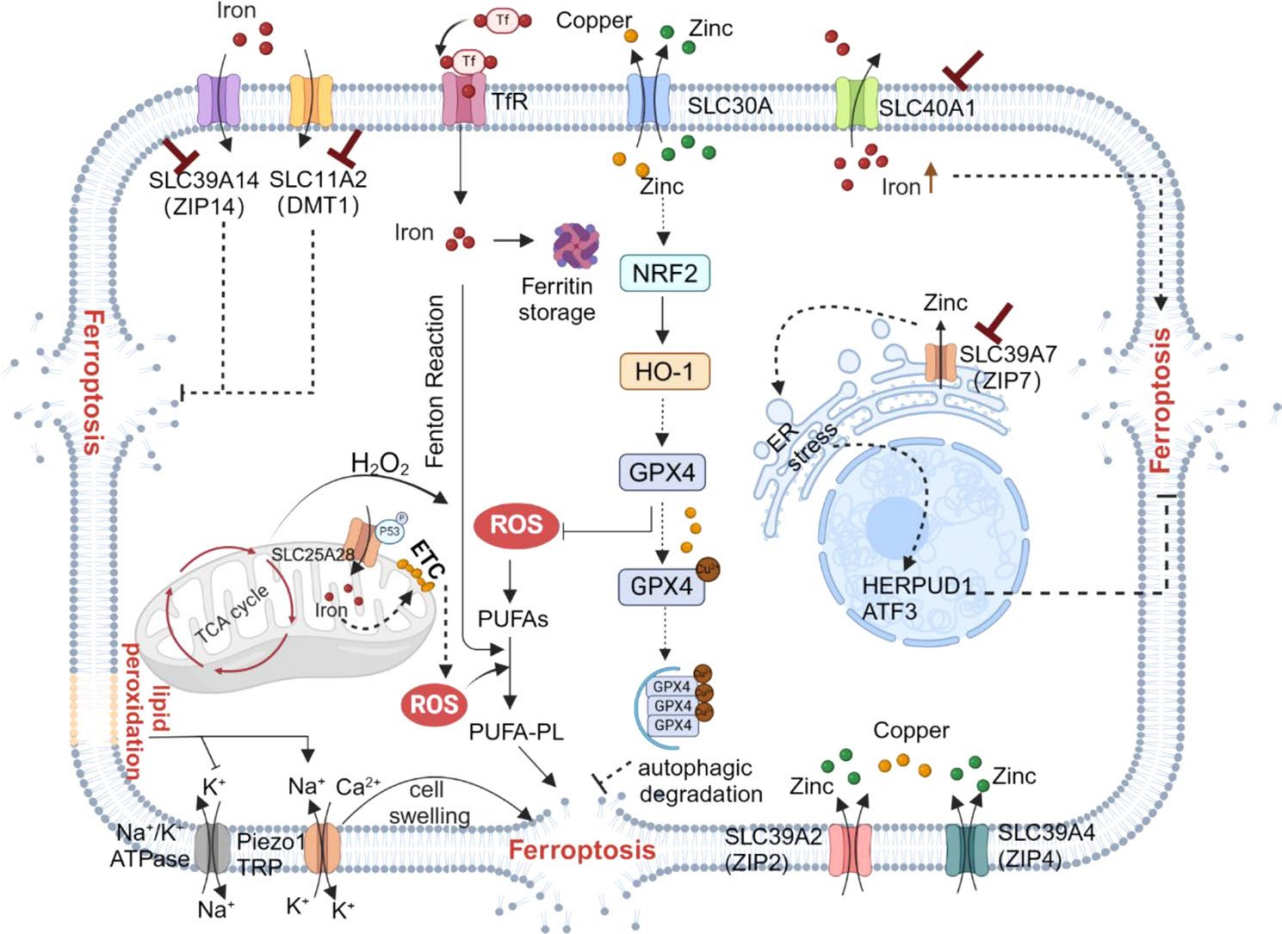
Regulation of metal-associated SLC transporters in ferroptosis. Fe^3+^ binds to TF and enters the cell via TFR1; it is then reduced and released into the labile iron pool (LIP) in the cytoplasm, where excess iron is stored as ferritin. LIP mainly exists in the form of Fe^2+^. Owing to the instability and high reactivity of Fe^2+^, iron produces hydroxyl radicals through the Fenton reaction; these directly react with PUFAs in the cell membrane and plasma membrane to produce large amounts of ROS, leading to ferroptosis. SLC40A1 is an iron-regulatory transporter that promotes the release of iron from cells. Abnormal expression of SLC40A1 can cause disorders of iron metabolism, lead to intracellular iron overload, and induce ferroptosis. Inhibition of DMT1/SLC39A14 (ZIP14) has been shown to attenuate ferroptosis. SLC25A28 interacts with mitochondrial p53 to induce ferroptosis by promoting the accumulation of redox-active iron and activation of the electron transport chain (ETC). Zinc can enhance NRF2/HO-1 expression, leading to increased levels of GPX4, SOD, and GSH, while decreasing lipid peroxide and ROS levels. Inhibition of ZIP7 prevents ferroptosis by upregulating endoplasmic reticulum stress-induced genes *HERPUD1* and *ATF3*. Copper has been shown to enhance ferroptosis-mediated tumor suppression by inducing autophagic GPX4 degradation. Membrane rupture is a key event in ferroptosis. Phospholipid peroxidation causes changes in ion flux (e.g., increased piezo-mediated Ca^2+^ uptake and TRP-mediated Ca^2+^ and Na^+^ uptake) and reduced Na^+^/K^+^ ATPase-mediated Na^+^ export and K^+^ uptake. These changes accelerate cell swelling and membrane rupture. Image created with BioRender

The importance of iron in ferroptosis is implicit in the definition of the process. Indeed, iron participates in ferroptosis of cells in various ways. For instance, iron chelators block ferroptosis, whereas exogenous iron increases the sensitivity of cells to ferroptosis. Moreover, SLC family members are essential for various aspects of iron metabolism, including intake, utilization, storage, and excretion (Fig. 5). For example, Fe^2+^ in the body can be transported into the cytoplasm via DMT1 (SLC11A2) or metal transporters (ZIP14 or 8) [104]. The SLC39/ZIP family acts as a broad metal ion transporter that mediates the uptake of a variety of important divalent metals including iron, but also zinc and manganese [105]. Solute carrier SLC39A14 (ZIP14) mediates iron uptake by transporting non-TF-bound iron across membranes. Conditional knockout of hepatic SLC39A14 in mice promotes hepatic injury in ferroptotic disease [106], whereas blocking YAP degradation suppresses Newcastle disease virus-induced ferroptosis by suppressing the expression of ZIP14 [107]. In addition, inhibition of SLC11 family member DMT1 has been shown to reduce iron accumulation and lipid peroxidation, thereby attenuating ferroptosis in rats with subarachnoid hemorrhage [108]; DMT1 silencing was also found to increase deacetylation of IDH2 by SIRT3 and GSH production to reduce erastin-induced ferroptosis [109]. In CuCP nanoparticle-induced ferroptosis, TfR1 and DMT-1 are upregulated in cancer cells to support intracellular Fe^2+^ accumulation and ferroptosis occurrence [110].

Mitochondria have notably high concentrations of iron, which is primarily involved in iron-sulfur clusters and heme synthesis. In addition, a pool of free and redox-active iron is present in mitochondria and has been shown to have a role in accumulation of mitochondrial ROS [111]. Mitoferrins, SLC25A family transporters localized to the inner mitochondrial membrane, are responsible for the entry of iron into mitochondria. While Mitoferrin-1 (SLC25A37) is highly enriched in erythrocytes, whereas mitoferrin-2 (SLC25A28) is widely expressed in various tissues. SLC25A28 interacts with mitochondrial p53 to induce ferroptosis by promoting the accumulation of redox-active iron and activation of electron transport chains [112]. However, it is not yet clear whether SLC25A37 similarly induces ferroptosis. However, it is not yet clear whether SLC25A37 similarly induces ferroptosis. SLC40A1, an iron-regulated transporter facilitating cellular iron release, has recently been linked to mediating streptozotocin-induced ferroptosis in type I diabetes mellitus [113].

Recently, evidence has emerged of associations of various other metal ion in ferroptosis, including Zn^2+^ and Cu^2+^. Zinc, an essential trace element, has crucial roles in human physiological and biochemical processes, and dysregulation of the zinc transporters is associated with several diseases. Regarding ferroptosis, zinc can enhance NRF2/HO-1 expression, leading to increased levels of GPX4, SOD, and GSH, while decreasing levels of lipid peroxides and ROS, which are pre-requisites for triggering ferroptosis [114]. Zinc is also related to SLCs, as it its transport across cell compartments is primarily controlled by two families of transporter members, SLC39A and SLC30A. The SLC39A family regulates zinc influx from the extracellular space or intracellular stores such as the endoplasmic reticulum into the cytosol, whereas the SLC30A family facilitates zinc efflux from the cytosol. SLC39A7 (ZIP7) has been identified as a key player in ferroptosis by a genome-wide RNA interference screen of zinc-related genes. Inhibition of ZIP7 prevents ferroptosis by upregulating endoplasmic reticulum stress-induced genes *HERPUD1* and *ATF3*, whereas zinc supplementation eliminates this protective effect. These findings provide evidence that zinc and ZIP7 are crucial regulators of ferroptosis [115].

The roles of SLC30A family members in ferroptosis and mediation of zinc transport remain to be further investigated. However, SLC30A family members are known to function as copper transporters, serving as the primary pathway by which eukaryotes (from yeast to humans) take up copper. SLC39A2 (ZIP2) and SLC39A4 (ZIP4) have also been found to transport copper in *Arabidopsis* [116]. In pancreatic cancer mouse models, copper has been shown to enhance ferroptosis-mediated tumor suppression by inducing autophagic GPX4 degradation. Conversely, copper chelators attenuated experimental acute pancreatitis associated with ferroptosis [117]. Further evidence is needed to determine whether the copper transporter regulates ferroptosis like the zinc transporter.

Plasma membrane disruption caused by accumulation of phospholipid peroxides is a key event in ferroptosis, and its effects are mediated by various ion. Phospholipid peroxidation causes changes in ion flux (e.g., increased piezo-mediated Ca^2+^ uptake, increased transient receptor potential (TRP)-mediated Ca^2+^ and Na^+^ uptake, and reduced Na^+^/K^+^ ATPase-mediated Na^+^ export and K^+^ uptake) and water entry, as well as exerting biophysical effects on membranes [118, 119]. Cell swelling and increased membrane stiffness further activate ion channels, such as Piezo and TRP, in a positive feedback manner, accelerating plasma membrane breakdown.

Trace metal ion also play important parts in nutrient metabolism, after entering cells through transporters on the cell membrane. Once inside the cell, these metal ion undergo metabolic processes catalyzed by various enzymes to produce energy and build biomolecules. Iron, copper, and zinc are particularly important in various metabolic pathways, including scavenging of intracellular free radicals. That ferroptosis is caused by peroxide free radicals attacking lipid molecules and oxidizing them to lipid peroxides, with the involvement of iron ion demonstrates the intricate connections among trace metal ion, metal ion transporters, and this form of cell death.

## Other ion-transporting SLCs and ferroptosis

**Fig. 6:**
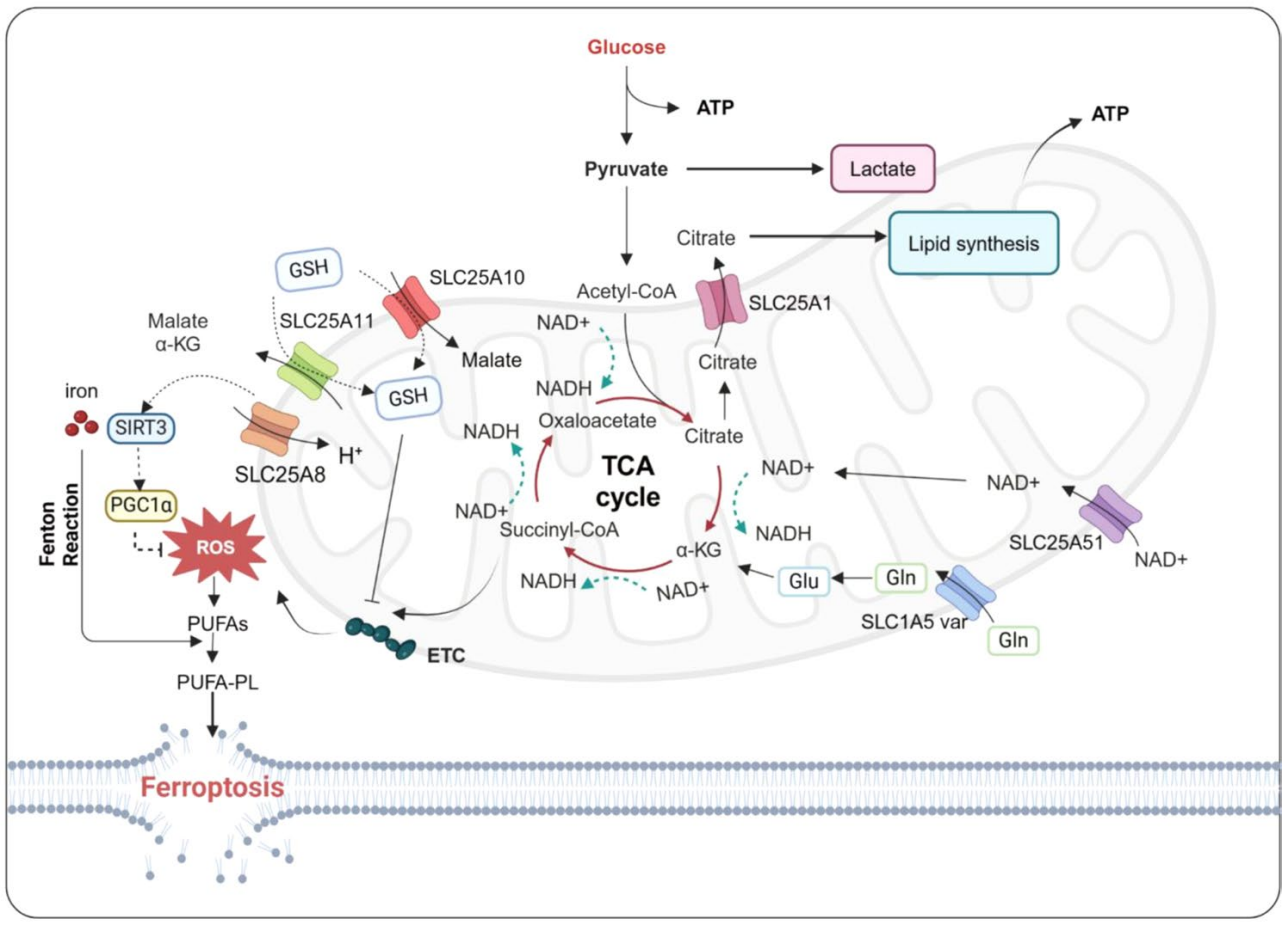
Regulation of ion-related SLC transporters in ferroptosis. Inhibition of SLC25A10 and SLC25A11 can exacerbate ferroptosis by inducing mitochondrial ROS, membrane depolarization, and depleting glutathione. Activation of SLC25A8 (UCP2)/SIRT3/PGC1α can inhibit excessive activation of ferroptosis and reduce ROS production. Loss of SLC25A51 expression affects mitochondrial oxygen consumption and ATP production and the transport of NAD^+^ into the mitochondrial matrix. NAD(P)^+^/NAD(P)H is crucial in ferroptosis. NAD(P)H can be utilized by NAD(P)H oxidoreductases to produce hydrogen peroxide, which then oxidizes membrane phospholipids containing PUFAs through the Fenton reaction, ultimately inducing ferroptosis. Image created with BioRender

Mitochondria have a crucial role in regulating oxidative phosphorylation, which is dependent on the activation of pyruvate dehydrogenase complex (PDHC) and carnitine palmitoyl transferase IA (CPTIA) [104, 111]to generate energy for TCA cycle. Various members of the SLC25 family support oxidative phosphorylation, including SLC25A10 and SLC25A11, which are ion channels responsible for transporting dicarboxylates (malonate, malate, and succinate) across the inner mitochondrial membrane in exchange for phosphate, sulfate, and thiosulfate (SLC25A11) or 2-oxoglutarate (SLC25A10). These transporters are thus essential to TCA cycling and metabolic processes including fatty acid synthesis. Inhibition of SLC25A10 and SLC25A11 in cardiomyocyte H9c2c cells has been shown to exacerbate ferroptosis by inducing mitochondrial ROS production, membrane depolarization, and depletion of glutathione [120](Fig. 6). Mitochondrial uncoupling protein 2 (UCP2, SLC25A8) is a member of the larger family of mitochondrial anion carrier proteins. Activation of the UCP2/SIRT3/PGC1α pathway can inhibit excessive activation of ferroptosis and reduce ROS production [66]. Mitochondrial citric acid transporter SLC25A1 facilitates the transport of citric acid between the mitochondrial matrix and cytosol. In addition, SLC13A5, which is anchored to the plasma membrane, enhances citrate uptake by hepatocytes from the blood [25]. SLC25A51 is also responsible for mediating the transport of nicotinamide adenine dinucleotide (NAD^+^) to mitochondria. Thus, loss of SLC25A51 expression significantly affects mitochondrial oxygen consumption and ATP production, as well as the transport of NAD^+^ into the mitochondrial matrix. Mitochondrial NAD^+^ transport deficiency disrupts oxidative phosphorylation and may reduce cell survival. The transition between NAD(P)^+^/NAD(P)H is also crucial in ferroptosis. Oxidoreductases such as NADPH-cytochrome P450 reductase (POR) and NADH^−^ cytochrome b5 reductase (CYB5R1) transfer electrons from NAD(P)H to oxygen, leading to the production of hydrogen peroxide; the hydrogen peroxide then oxidizes membrane phospholipids containing PUFAs through the Fenton reaction, ultimately inducing ferroptosis [121]. The basal level of NADPH can serve as a biomarker for the susceptibility of cancer cell lines to ferroptosis, as NADPH deficiency promot cell ferroptosis. However, NADPH can also be utilized by NADPH oxidase to generate superoxide anions, and activation or inhibition of this process may also indicate whether cells are approaching the irreversible stage of ferroptosis [38].

## SLCs as clinical therapeutic targets

**Fig. 7:**
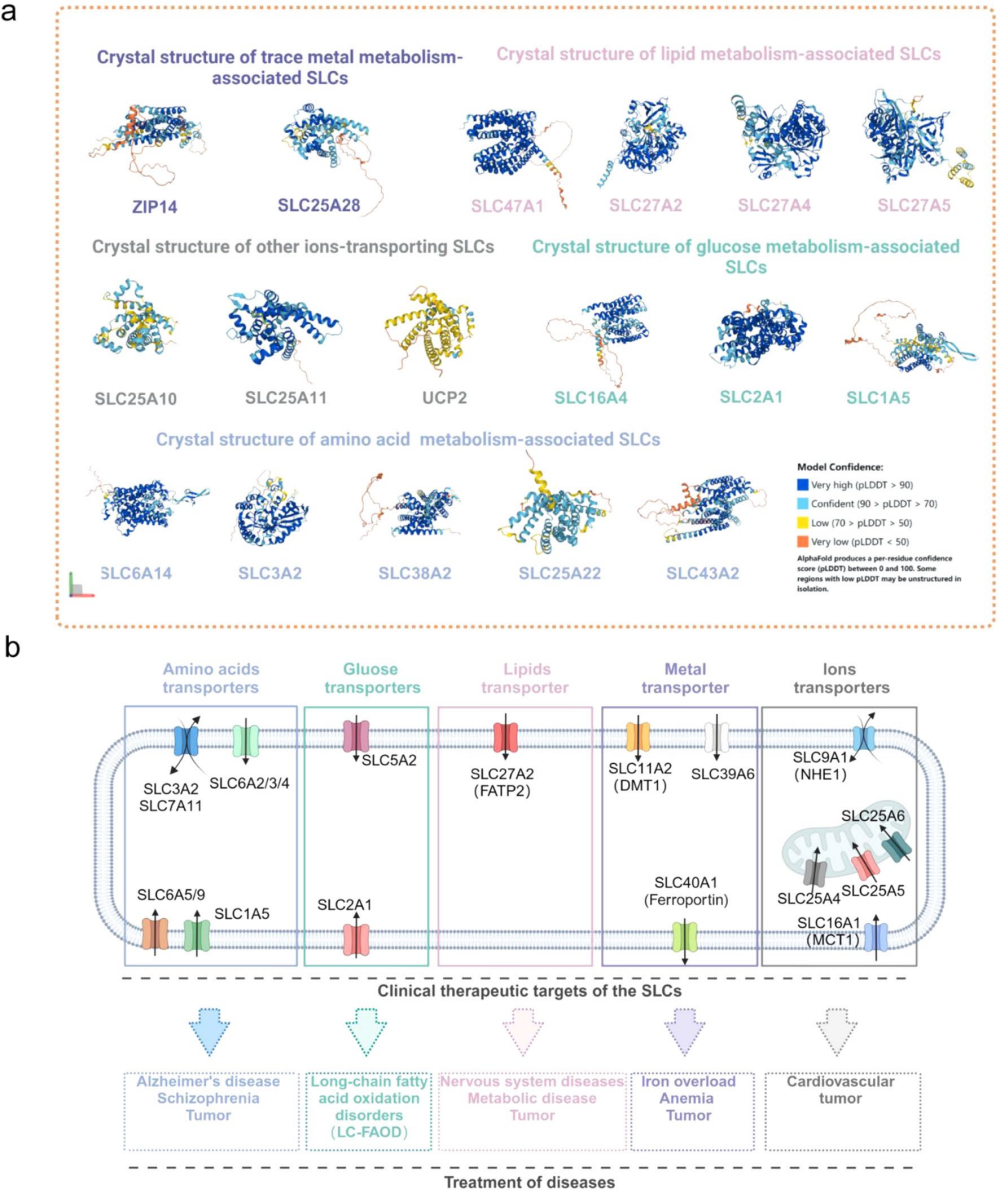
Clinical therapeutic targets of SLC transporters in a variety of diseases. (**a**) Crystal structures of SLCs associated with ferroptosis, from the UniProt database (https://www.uniprot.org/). (**b**) Potential targets of SLCs associated with amino acid, glucose, lipids, metals, and other ion that are or could be used to treat a variety of diseases. Image created with BioRender

SLCs are widely expressed in cell membranes and organelle membranes, and their abnormalities have been implicated in a variety of diseases including single-gene disorders, neurodegenerative diseases, tissue and organ damage, immune-related diseases, and tumors. Therefore, SLC are emerging drug targets. In this section, we map the crystal structures of ferroptosis-associated SLCs (Fig. 7a) and summarize the clinical therapeutic targets of five classes of ferroptosis-related SLC transporters in various diseases (Fig. 7b). In addition, we summarize the dozen classes of inhibitors or clinical drugs that can directly modulate SLC function(Table 3).Current research on SLC family chemical probes focuses primarily on monoamine transporters (SLC6A2/3/4), glucose transporters (SLC5A2, SLC2A1), and glycine transporters (SLC6A5/9). Monoamine reuptake inhibitors that target SLC6A2/3/4 are the largest class of therapeutic drugs aimed at SLC transporters, with more than 40 such molecules approved by the US Food and Drug Administration (FDA) as inhibitors of neurotransmitter reuptake [103]. Glucose transporters SLC2A and SLC5A are highly expressed to in tumor cells to maintain an increased energy supply. Several therapeutic drugs are available to target glucose transporters; these include diuretics (bumetanide), antidepressants (selective serotonin reuptake inhibitors), and SGLT-2 inhibitors [122]. Triheptanoin, an FDA-approved drug targeting GLUT1 (SLC2A1), is used to treat long-chain fatty acid oxidation disorders (LC-FAOD) [123]. Drugs such as VVZ-149, BI 425,809, and PF–03463275, which target SLC6A5/9, are being investigated with respect to their applications in postoperative pain management and treatment of schizophrenia and Alzheimer’s disease and have entered phase I clinical trials [103]. Tumor cells adapt to an acidic environment through pH regulation of transmembrane ion transporters, and intracellular pH is regulated by synergistic action of several transporters (SLC4/26 for transport of HCO_3_^−^, SLC9A1 for transport of Na^+^ and H^+^, and SLC16 for transport of monocarboxylic acids). Although inhibitors of these transporters have shown significant antitumor effects in preclinical experiments, their effectiveness in clinical trials for cancer treatment has not been satisfactory; examples include inhibitors of SLC9A1, cariporide, eniporide, and zoniporide [124]. However, AZD3965, an inhibitor of SLC16A1 (MCT1), is a notable new drug that is currently undergoing phase III clinical trials in cancer patients [103].

**Table 3:** Ferroptosis-associated SLCs targeted by (pre) clinical drugs or inhibitors

Category	Gene name	Inhibitors/Drugs name		Disease/symptom	Refs.
Glucose metabolism-associated SLCs	SLC5A2		Dapagliflozin, Canagliflozin, Ertugliflozin, Ipragliflozin, Phloretin, etc.	Type II Diabetes, Rheumatoid arthritis	[103]
	SLC2A1		Triheptanoin	LC-FAOD	[123]
Lipid metabolism-associated SLCs	SLC27A2		Lipofermata	N/A	[130]
Amino acid metabolism-associated SLCs	SLC6A2/3/4		**MRIs**: citalopram, escitalopram, fluoxetine, fluvoxamine, paroxetine, sertraline, desvenlafaxine, duloxetine, levomilnacipran, milnacipran, atomoxetine, bupropion dexmethylphenidate, benztropine	Major Depression Disorder (MDD), Anxiety Disorders, Obsessivecompulsive disorder (OCD), Attention-deficit hyperactivity disorder (ADHD), NarcolepsyNeurological Disorder	[103]
	SLC6A5/9		VVZ-149, BI 425,809, and PF-03463275	Postoperative pain, Schizophrenia and Alzheimer’s disease	[103]
	SLC1A5		V9302	Tumor	[129]
	system Xc- (SLC7A11 and SLC3A2)or system L(SLC7A5 and SLC3A2)		erastin, IKE, morpholine erastin III, piperazine erastin, Aldehyde erastin, Sulfasalazine, Sorafenib, and YT521-B homolog 2(YTHDC2)	Tumor, Rheumatoid arthritis, Ulcerative colitis, etc.	[79, 127, 128]
Trace metal metabolism-associated SLCs	SLC40A1		LY2928057, VIT2763	Anemia and iron overload	[104]
	SLC39A6/LIV-1		Ladiratuzumab Vedotin	Metastatic and triple-negative breast cancer	[104]
Other ion-transporting SLCs	SLC16A1		AZD3965	Cancer	[103]
	SLC25A4/5/6		Clodronate	Osteoporosis	[103]
	SLC9A1		Cariporide, Eniporide, Zoniporide, Rimeporide	Coronary heart disease, Cardiovascular diseases, Schizophrenia, Seerebipolar disorder and Duchenne muscular dystrophy	[103, 124]

As early as 2003, before ferroptosis had been formally defined, SLC7A11 was found to act as a direct target of erastin, a small-molecule inducer of ferroptosis [125]. Then, in 2008, compound RSL3 was shown to induce a non-apoptotic and iron-dependent form of oxidative cell death in vitro and in vivo [120], which was later defined as ferroptosis. Since then, ferroptosis has garnered significant research attention. A growing body of evidence suggests that targeting ferroptosis could open new avenues for the treatment of various diseases, particularly cancers and neurodegenerative diseases. However, although RSL3 has been identified as direct inhibitor of GPX4, which is an essential factor for cell survival, its in vivo and clinical applications remain unfeasible owing to its low solubility and poor pharmacokinetics [126].

Targeting SLCs in combination with ferroptosis inducers is a potential therapeutic approach for multiple diseases. For instance, numerous studies have demonstrated that targeting cystine transporter SLC7A11 is a potential means of inducing ferroptosis for therapeutic purposes, either alone or in combination with other drugs, radiotherapy, or immunotherapy, with particular promise for the treatment of cancers and neurodegenerative diseases. Inhibitors of system Xc^−^, such as erastin, IKE, glutamate, morpholine erastin III, piperazine erastin, aldehyde erastin, sulfasalazine, sorafenib, and YTHDC2, have been reported to enhance therapeutic effects in multiple diseases by directly or indirectly inhibiting the activity of system Xc^−^ (comprising SLC3A2 and SLC7A11) and/or system L (comprising SLC3A2 and SLC7A5) [79, 127, 128]. V-9302 is a competitive antagonist that targets amino acid transporter SLC1A5 (ASCT2), inhibiting transmembrane glutamine flux selectively and potently. Pharmacological inhibition of SLC1A5 by V9302 has been shown to improve the efficacy of anti-PD-1 therapy [129]. Another inhibitor, Lipofermata (targeting FATP2), has been demonstrated to reduce or prevent tumor growth when combined with a drug that interferes with cell division. Combining inhibition of FATP2 with anti-CTLA4 and anti-CSF1R antibodies has also shown positive anti-tumor effects [130].

Furthermore, metal ion transporters have essential roles in the regulation of ferroptosis, particularly those that regulate intracellular iron levels. Divalent metal transporter 1 (DMT1/SLC11A2) is associated with intracellular iron levels and iron homeostasis, and recent studies have shown that temozolomide, a first-line clinical agent for treatment of glioblastoma, can induce ferroptosis to inhibit cell growth by targeting DMT1 in glioblastoma [131]. Furthermore, FPN1 (SLC40A1) mediates ferroptosis by regulating intracellular iron homeostasis. The drugs LY2928057 and VIT2763, which inhibit SLC40A1/FPN1, have been investigated in phase I clinical studies for the treatment of anemia and iron overload [104]. Furthermore, zinc has been found to boost GPX4 expression and increase GSH levels, preventing ferroptosis by reducing levels of lipid peroxides and ROS. Zinc ion transporter SLC39A7/ZIP7, which is responsible for maintaining endoplasmic reticulum homeostasis, has been identified as a key genetic determinant of ferroptosis; this finding has significant therapeutic implications for diseases involving ferroptosis and zinc imbalances. Another zinc transporter, SLC39A6, has emerged as a cancer-associated factor [132]. Ladiratuzumab vedotin, an antibody–drug conjugate that targets SLC39A6/LIV-1, is currently undergoing clinical research as a treatment for metastatic and triple-negative breast cancer [104]. Mitochondria, as the main source of cellular ROS, have an important role in ferroptosis [133, 134]. Mitochondrial membrane transporters of the SLC25 family (SLC25A4, SLC25A5, and SLC25A6) convert ADP to ATP in mitochondrial proton output. Clodronate leads to accumulation of intramitochondrial protons and hyperpolarization of the inner mitochondrial membrane, resulting in loss of mitochondrial function by inhibiting ADP/ATP conversion [124]. Targeting mitochondrial solute transporters to induce mitochondrial dysfunction in cancer cells could be a potential approach for anti-tumor therapies in the future. All these findings indicate that the combination of SLC inhibitors with ferroptosis inducers is a promising therapeutic strategy.

## Conclusion

In the 10 years since the discovery of ferroptosis, efforts have been made to address the key mechanisms that regulate this novel form of cell death. Although GSH–GPX4 is the most widely recognized ferroptosis inhibition system, recent years have seen the gradual discovery of GPX4-independent mechanisms of ferroptosis inhibition, involving factors such as FSP1-CoQ10, GPX4-DHODH, GCH1/BH4-LPs, and 7-DHC-DHC7R. Rupture of the cell membrane is key to the eventual occurrence of ferroptosis; however, the mechanism by which this takes place remains unclear. Most researchers believe that lipid peroxidation due to iron overload leads to membrane rupture via changes in membrane fluidity and permeability, although Michael Overholtzer has suggested that membrane rupture in ferroptosis is mediated by formation of nanoscale plasma membrane pores [50, 135]. Recently, it was reported that NINJ1, the executive factor in plasma membrane breakdown associated with pyroptosis, is oligomerized during ferroptosis, and NINJ1 deficiency can protect macrophages and fibroblasts from ferroptosis-related membrane breakdown [136]. In addition, NINJ1 has been found to interact with system Xc^−^ (comprising members of the SLC family), which is responsible for cystine uptake. After NINJ1 knockdown, system Xc^−^ levels and stability were increased, cystine uptake was enhanced, and CoA and GSH levels were increased; these effects collectively provided protection against ferroptosis [137]. The most numerous transmembrane protein family after G protein-coupled receptors, SLCs exist in cell membranes and a variety of organelle plasma membranes and are widely involved in the transport of intracellular carbohydrate, fat, and amino acid; moreover, some SLC family members are known to participate in induction or inhibition of ferroptosis. Ferroptosis is also affected by various metabolic pathways such as amino acid, lipid, and sugar metabolism. Targeting ferroptosis via induction and inhibition of nutrient metabolic pathways is a promising approach for the treatment of drug-resistant cancers, ischemic organ damage, and degenerative diseases associated with lipid peroxidation [10]. However, more studies are required to determine whether more susceptibility factors for ferroptosis exist within the SLC family and whether SLCs participate in the occurrence of cell membrane rupture in ferroptosis.

## Data Availability

No datasets were generated or analysed during the current study.

## Abbreviations

AA: Arachidonoyl
AdA: Adrenoyl
ADC: Antibody-drug conjugate
AKI: Acute kidney injury
ALOXs: Arachidonate lipoxygenase
CAC: Carnitine acyl-carnitine carrier
CAT: Capsiate ester
CoQH2: Ubiquinol
ETC: Electron transport chains
FABP(pm): The plasma membrane-associated Fatty acid-binding protein
FATPs: Fatty acid transporters
HGNC: The Human Genome Organization Gene Nomenclature Committee
HUGO: The Human Genome Organization
LD: Lipid droplets
LIP: Labile iron pool
LPO: Lipid peroxides
MCR: Methionine/cystine restriction
MCT: Monocarboxylic acid transporter
MUFAs: Monounsaturated fatty acids
NAD^+^: The nicotinamide adenine dinucleotide
OXPHOS: Oxidative phosphorylation
PDAC: Pancreatic ductal adenocarcinoma
PDHC: Pyruvate dehydrogenase complex
PLOOHs: Phospholipid hydroperoxides
PMN-MDSCs: Polymorphonuclear myeloid-derived suppressor cells
PUFA-CE: PUFA esterified cholesterol esters
PUFAs: Polyunsaturated fatty acids
ROS: Reactive oxygen species
SLC: The solute carrier transporters
TCA: Tricarboxylic acid
TF: Transferrin
TfR: Transferrin receptor
TME: The tumor microenvironment
α-KG: Alpha-ketoglutarate
